# Neuroprotective Mechanisms of Three Natural Antioxidants on a Rat Model of Parkinson’s Disease: A Comparative Study

**DOI:** 10.3390/antiox9010049

**Published:** 2020-01-06

**Authors:** Lyubka P. Tancheva, Maria I. Lazarova, Albena V. Alexandrova, Stela T. Dragomanova, Ferdinando Nicoletti, Elina R. Tzvetanova, Yordan K. Hodzhev, Reni E. Kalfin, Simona A. Miteva, Emanuela Mazzon, Nikolay T. Tzvetkov, Atanas G. Atanasov

**Affiliations:** 1 Department of Behavior Neurobiology, Institute of Neurobiology, Bulgarian Academy of Sciences, Sofia 1113, Bulgaria; stela_dragomanova@abv.bg (S.T.D.); saalexandrova@gmail.com (S.A.M.); 2 Department of Synaptic Signaling and Communications, Institute of Neurobiology, Bulgarian Academy of Sciences, Sofia 1113, Bulgaria; m.lazarova@gmail.com (M.I.L.); reni_kalfin@abv.bg (R.E.K.); 3 Department Biological Effects of Natural and Synthetic Substances, Institute of Neurobiology, Bulgarian Academy of Sciences, Sofia 1113, Bulgaria; aalexandrova@abv.bg (A.V.A.); elina_nesta@abv.bg (E.R.T.); 4 Department of Pharmacology, Toxicology and Pharmacotherapy, Faculty of Pharmacy, Medical University, Varna 9002, Bulgaria; 5 Department of Biomedical and Biotechnological Sciences, University of Catania, Via S. Sofia 89, 95123 Catania, Italy; ferdinic@unict.it; 6 Department of Sensory Neurobiology, Institute of Neurobiology, Bulgarian Academy of Sciences, Sofia 1113, Bulgaria; jordanqvo@gmail.com; 7 IRCCS Centro Neurolesi “Bonino-Pulejo”, Via Provinciale Palermo, Contrada Casazza, 98124 Messina, Italy; emanuela.mazzon@irccsme.it; 8 Department of Biochemical Pharmacology and Drug Design, Institute of Molecular Biology, Bulgarian Academy of Sciences, Sofia 1113, Bulgaria; ntzvetkov@gmx.de; 9 Department of Molecular Biology, Institute of Genetics and Animal Breeding of the Polish Academy of Sciences, Jastrzebiec, 05-552 Magdalenka, Poland; 10 Department of Pharmacognosy, University of Vienna, 1090 Vienna, Austria; 11 Ludwig Boltzmann Institute for Digital Health and Patient Safety, Medical University of Vienna, Spitalgasse 23, 1090 Vienna, Austria

**Keywords:** ellagic acid, α-lipoic acid, myrtenal, rat, Parkinson’s disease, neuroprotection, antioxidant activity, dopamine

## Abstract

We compared the neuroprotective action of three natural bio-antioxidants (AOs): ellagic acid (EA), α-lipoic acid (LA), and myrtenal (Myrt) in an experimental model of Parkinson’s disease (PD) that was induced in male Wistar rats through an intrastriatal injection of 6-hydroxydopamine (6-OHDA). The animals were divided into five groups: the sham-operated (SO) control group; striatal 6-OHDA-lesioned control group; and three groups of 6-OHDA-lesioned rats pre-treated for five days with EA, LA, and Myrt (50 mg/kg; intraperitoneally- i.p.), respectively. On the 2nd and the 3rd week post lesion, the animals were subjected to several behavioral tests: apomorphine-induced rotation; rotarod; and the passive avoidance test. Biochemical evaluation included assessment of main oxidative stress parameters as well as dopamine (DA) levels in brain homogenates. The results showed that all three test compounds improved learning and memory performance as well as neuromuscular coordination. Biochemical assays showed that all three compounds substantially decreased lipid peroxidation (LPO) levels, and restored catalase (CAT) activity and DA levels that were impaired by the challenge with 6-OHDA. Based on these results, we can conclude that the studied AOs demonstrate properties that are consistent with significant antiparkinsonian effects. The most powerful neuroprotective effect was observed with Myrt, and this work represents the first demonstration of its anti-Parkinsonian impact.

## 1. Introduction

Parkinson’s disease (PD) is the second most common progressive neurodegenerative disorder after Alzheimer’s disease (AD) and as such represents a serious social burden. It is characterized by selective loss of dopaminergic neurons from *substantia nigra pars compacta* (SNPC) and their projections to the striatum [1,2,3]. Impairment of motor function and memory are the primary clinical characteristics of PD observed both in patients and experimental animal models.

Several lines of independent data convergently indicate that dysregulated oxidative responses play a major role in disease onset, maintenance, and progression [4,5,6]. One of the major sources of reactive oxygen species (ROS) in SNPC may originate from changed DA metabolism [7]. Studies in patients with PD showed increased levels of oxidized lipids, proteins, and DNA, as well as decreased levels of reduced glutathione (GSH) [8] in SNPC. Consequences of OS are intracellular accumulation and extracellular release of proteins with abnormal conformation, which then penetrate into adjacent neurons, thus stimulating the neurodegenerative progression [9].

Current therapy for the disease is symptomatic and fails to influence the course of the disease and its progression [10,11].

Hence, as PD remains largely an unmet medical need, obtaining a better understanding of the pathogenic mechanisms operating in PD development, including the dysregulated OS responses strongly warrant discovery of novel pathogenic-tailored approaches for the treatment of the disease. One potential approach to the discovery of new therapeutic opportunities relies on the development of medications based on naturally occurring substances.

**Figure 1:**
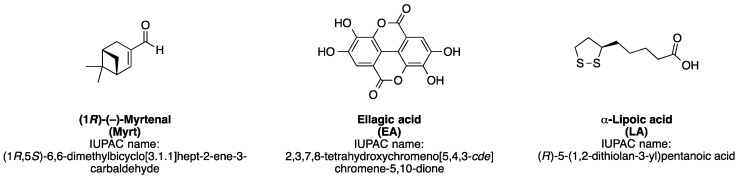
Chemical structures and IUPAC (International Union of Pure and Applied Chemistry) names of myrtenal, ellagic acid, and alpha-lipoic acid.

Along this line of research, we presently examine three natural bio-antioxidants (AOs), namely the plant monoterpene myrtenal (Myrt), the polyphenol ellagic acid (EA), and the natural dithiol alpha-lipoic acid (LA). They all are strong bio-antioxidants, which have shown promising preclinical and clinical effects in several pathological conditions (for structures of compounds, see Figure 1) [12,13].

LA and EA derivatives and some supplements representing a rich source of both have been tested on humans, and have improved some symptoms that also occur in PD. The administration of LA in combination with omega-3 fatty acids slowed cognitive and functional decline in a 12 month trial in AD patients [14]. Another study showed significant improvement of total antioxidant capacity in LA-treated multiple sclerosis patients in comparison to non-treated ones after 12 weeks [15]. EA, on the other hand, restored cognitive performance related to mild age-related declines in overweight participants aged around 50 years after 12 weeks EA treatment course [16]. Supplementation with EA-rich foods and beverages as pomegranate juice has been shown to improve the antioxidant status in aged healthy humans (aged over 60 years) after four weeks of treatment [17,18].

To our knowledge, none of these substances has been applied in clinical trials on PD patients to date, and there are only scarce in vivo or in vitro data for ameliorating disease effects for some of them.

EA (2,3,7,8-tetrahydroxychromeno[5,4,3-*cde*]chromene-5-10-dione) is the substance mainly contributing to the various and notorious beneficial effects of *Punica granatum* L. (pomegranate) extracts. EA is also widely available in most berries and nuts [19,20,21].

Of particular interest for this study is the reported ability of EA to lower the rotenone-induced generation of reactive oxygen species (ROS) and reactive nitrogen species (RNS) in PC12 cells [22] as well as its ability to exhibit a neuroprotective effect against oxidative damage in diabetic rats by suppressing oxidative stress [23]. From cognitive and behavioral points of view, EA prevents scopolamine- and diazepam-induced cognitive impairments [24,25] and may be of potential benefit in brain stroke by alleviating the oxidative stress characteristic for this condition [26]. Data also supports EA’s brain-protective properties against 6-OHDA-induced neuroinflammation in rats as well as cognitive and long-term potentiation deficits following traumatic brain injury [27].

EA alleviates oxidative damage through several mechanisms: (1) activation of antioxidant response using the Nrf2 (nuclear erythroid factor 2) [28,29]; (2) inhibition of cyclooxygenase 2 (COX-2) [30] and cytokines regulated through NF-kB (nuclear factor-kappa (B) [31,32]; (3) modulation of cell survival and/or apoptosis [33]; and (4) potentiation of biological antioxidants and antioxidant enzymes activity [34].

LA ((*R*)-5-(1,2-dithiolan-3-yl)pentanoic acid) is a naturally occurring substance, a dithiol that plays fundamental role in mitochondrial metabolism as a coenzyme for pyruvate dehydrogenase and alpha-ketoglutarate dehydrogenase. It is also a substrate for the NADPH-dependent enzyme glutathione reductase. According to present understanding, LA is most likely synthesized in human and animal mitochondria [35]. It is not considered to be a vitamin but is able to affect the levels of some vitamins in the organism [36]. Its protective in vivo and in vitro activities against a range of pathophysiological conditions have been reported [37], including protection against MPP^+^-induced toxicity in neuronal cells [38]. 

LA has been widely used in clinical setting as a supplementary treatment for different conditions associated with increased oxidative stress. Several studies have shown that LA exerts protective effects in in vivo and in vitro experimental models of neurodegenerative diseases, including Alzheimer’s disease (AD), macular degeneration, and PD [39,40,41,42,43]. Administration in moderate doses has produced no evidence of serious side effects [38,44,45,46,47,48].

Myrtenal (1*R*,5*S*)-6,6-dimethylbicyclo[3.1.1]hept-2-ene-2-carboxaldehyde) is a bicyclic monoterpenoid established in many plant essential oils, including *Cuminum cyminum* [49] and *Lavandula* spp. [50]. Myrt demonstrates multiple biopharmacological activities in different experimental conditions. Accordingly, Myrt influences apoptotic and pro-apoptotic signaling pathways, stabilizes intrinsic antioxidant protection, suppresses TNF-α [51] expression, inhibits tumor growth, regulates the activity of number of lysosomal and mitochondrial enzymes, and influences the processes of gluconeogenesis in tumor cells [51,52,53]. This monoterpenoid is relatively poorly studied in the field of neuroscience. Our previous research demonstrated preventive effects of Myrt on Alzheimer’s type dementia and amnesia in ICR mice and Wistar rats, thus re-confirming the role of OS mechanisms in AD pathogenesis [40]. So far, there is no data in the literature about effects of Myrt on PD progression.

These multiple lines of evidence propelled us to investigate influence of these three natural AOs, namely EA, LA and Myrt, in a head-to-head comparison on the development of the neurodegenerative process of experimental PD induced by intrastriatal administration of 6-hydroxydopamine (6-OHDA).

## 2. Materials and Methods

### 2.1. Materials

6-Hydroxydopamine hydrochloride; (1*R*)-(-)-Myrtenal; *R*-(−)-Apomorphine hydrochloride hemihydrate; ethylenediaminetetraacetic acid (EDTA); dopamine hydrochloride; and chloral hydrate were purchased from Sigma-Aldrich (Schelldorf, Germany). Potassium iodide was purchased from Honeywell/Fluka (Munich, Germany). Ellagic acid hydrate was obtained from AlfaAesar (Kandel, Germany). α-Lipoic acid was purchased from Solupharm GmbH & Co. KG (Melsungen, Germany) as a Thiogamma Turbo-Set solution for injection at 600 mg and 50 mL.

### 2.2. Animals

Male Wistar rats (250–300 g) were used (Experimental and Breeding Base for Laboratory Animals, Dragomansko Shose Str. No 1, Slivniza 2200, Bulgaria). Animals were housed in plastic cages with free access to food and water and were maintained in a controlled environment (20 ± 2 °C, 50 ± 10% relative humidity, 12-h light/dark cycle). Each experimental group contained 8–10 animals. The experiments have been performed strictly according to the national regulations and European Communities Council Directive (86/609/EEC) as well as the “Principles of laboratory animal care” (NIH publication No. 85-23) concerning the protection of animals used for scientific and experimental purposes.

### 2.3. Experimental Protocol of the PD Model

Rats were anesthetized with chloral hydrate (400 mg/kg b. w., i. p.) [54], their heads were shaved and skin cleaned with 70% ethanol. Then the rats were positioned in the stereotaxic apparatus by their ear canals. A midline incision was done, the subcutaneous and muscle tissues were separated, and the bregma and lambda areas were cleaned. A thin hole was made in the skull at the appropriate location. The target coordinates for striatum were AP = 0; LR = 3.5; H = + 5 from the bregma, according to the stereotaxic atlas [55]. The lesion was made by injection of 2 µL (total 10 µg) 6-OHDA into the right striatum. The injection was conducted at a rate of 1 µL/min and needle was left in place for five min, before it was slowly taken back [56]. Finally, the incision was glued and animals returned to their cages to recover. The sham-operated group was subjected to all procedures, except that saline was injected instead of 6-OHDA.

### 2.4. Drug Treatment and Experimental Design

#### Animal Groups and Experimental Protocols

The rats were divided into the five following groups:

SO (sham operated, 2 µL saline in striatum and treated daily with 0.5 mL/100 g saline, i.p.),
6-OHDA (2 µL/10 µg 6-OHDA in striatum and treated daily with 0.5 mL/100 g saline, i.p.),
6-OHDA + EA (lesioned and treated daily with 50 mg/kg EA, i.p.),
6-OHDA + LA (lesioned and treated daily with 50 mg/kg LA, i.p.),
6-OHDA + Myrt (lesioned and treated daily with 50 mg/kg Myrt, i.p.).

Pretreatment was done for five days before the surgery. The doses of used compounds were chosen on the basis of our previous studies [25,40,43,57] as well as literature data [12,13,22,26,29,33,49,58,59]. Using the allometric calculation [60,61], a rat dose of 50 mg/kg (weight of 275 g male Wistar rats) of each compound (e.g., EA, LA or Myrt) would correspond to a human dose (human equivalent dose, HED) of approximately 9.8 mg/kg, which is near the clinical dose of amantadine (60 kg middle body human weight).

Preparation of the solutions was made in accordance with good laboratory practice (GLP) for the preparation of drug emulsions. After weighing the specified amount of Myrt or EA, an emulsion was prepared with emulsifier (lecithin for Myrt and Gumma Arabica for EA). The aqueous phase of the emulsion was saline. A homogenizer was used to prepare a stable emulsion daily ex tempore and administered (intraperitoneally for Myrt and via an intragastric probe for EA) to the indicated volumes in experimental rats. Alfa lipoic acid was used as a solution for injection (Thioctic acid 600 mg/ 24 mL, MEDA Phanna GmbH & Co. KG, Germany).

On the 2nd and 3rd week post lesion, animals were subjected to behavioral tests (apomorphine-induced rotations, rotarod test, and the passive avoidance test). Immediately after completion of the behavioral battery, the animals were euthanized for biochemical studies. Body weight of rats from each groups was measured on the first day (before treatment) of the experiments, after five days pretreatment and on the II and III weeks post lesion with 6-OHDA.

### 2.5. Behavioral Observations

#### 2.5.1. Apomorphine-Induced Rotation Test (Apo)

Drug-induced rotational behavior has conventionally been used to determine the extent of unilateral lesions. After a 10 min habituation period, each animal was injected with apomorphine (2 mg/kg, i.p.). Counting of full contralateral rotations (opposite to the lesion) started 1 min after injection and was monitored in a cylindrical container (with a diameter of 33 cm and a height of 35 cm) for 30 min in a dimly lighted room [62].

#### 2.5.2. Rotarod Test (RR)

The rotarod test is designed to evaluate alterations in motor coordination following administration of pharmacological agents causing either sedation or muscle relaxation [63]. The rotarod test was conducted using an apparatus consisting of a wooden rod with a non-slip surface. Before THE 6-OHDA lesion, rats were placed on the rotating rod (7 rpm/min) for training. Test session was made on the 2nd and 3rd week after surgery. The animals were placed on the rotating rod (7 rpm/min) and the number of falls/min was recorded.

#### 2.5.3. Learning and Memory (Passive Avoidance Test)

Learning and memory performances in the rats were evaluated using a passive avoidance learning test [64]. The apparatus chamber used in this test was composed by a black poorly illuminated compartment and a white illuminated compartment.

Acquisition/conditioning phase: during this phase, each animal was placed in the white compartment. When the rodent innately crossed to the black compartment, it received a mild electrical foot shock (0.5 mA, 1 s). This procedure allows the rat to learn that moving to the dark compartment had negative consequences and it therefore passively avoids stepping through. In this trial, the initial latency (IL, acquisition latency time) of entrance into the dark chamber was recorded, and rats with ILs > 60 s were excluded from the analysis. 

Test phase: On the 14th and 21st day post lesion, each rat was placed in the illuminated chamber for evaluation of passive avoidance response. The interval between the placement in the illuminated chamber and the entry into the dark chamber was measured as step through latency (STL) (up to a maximum of 180 s as cut-off).

All behavioral observations were carried out between 9 a.m. and 12 a.m. After the last behavioral tests, rats were euthanized by CO_2_, and their brains were quickly removed for biochemical analyses.

### 2.6. Neurochemical Determination of Dopamine

The frontal cortex and hippocampus were removed on ice and were kept frozen at −40 °C for approximately 24 h. The tissue samples were weighed and homogenized in 5 mL butanol and an appropriate amount of 0.01 N HCl. The homogenates were centrifuged at 1500 rpm for 10 min. A 2 mL aliquot of butanol supernatant was poured into the centrifuge tube containing 1.5 mL of 0.1 M phosphate buffer with a pH of 6.5. The mixture was stirred on a Vortex apparatus for 20 s. Thus DA was extracted into the phosphate buffer. The samples were then centrifuged at 3000 rpm for 10 min to separate the organic and aqueous layers, and the upper organic layer was aspirated using a vacuum with a liquid trap. Dopamine in phosphate extract was oxidized into fluorophores according to the method of Jacobowitz and Richardson [65]. During the fluorescence reaction to 1 mL of the phosphate phase, at two minute intervals, we added 0.25 mL of ethylenediaminetetraacetic (4.0 g EDTA diluted in 100 mL dH_2_O), pH 6–6.5; 0.2 mL iodide solution (4.8 g Potassium jodide + 0.25 g iodide crystals diluted in 100 mL dH_2_O); 0.25 mL alkaline sulfites (0.25 g Na_2_SO_3_ and 1 mL dH_2_O + 9 mL 4N NaOH and 0.3 mL 5N CH_3_COOH). Samples were run sequentially for 5 min in a boiling water bath and after that for 1 min in an ice-cold water bath. The fluorescence of DA was read at λ = 320/385 nm. DA levels were calculated based on the fluorescence of a standard DA solution and were expressed in picograms per gram of fresh tissue (pg/g tissue).

The microdialysis with HPLC detection today represents the gold standard for DA detection. However, in the present study, we used a 6-OHDA experimental model of Parkinson’s disease. Its hallmark is the stereotaxic administration of the toxin directly into the striatum, which slowly provokes progressive lesion of the nigrostriatal pathway. Striatal terminal damage started within the first day of 6-OHDA stereotaxic injection, whilst nigral cell loss takes a minimum of one week, reaching its maximum within 2–3 weeks [66,67,68]. Microdialysis technique, in turn, is also an invasive method, which is accompanied by damage to the brain tissue. If performed, the microdialysis technique should be the second consecutive invasive method used on the brain after stereotoxic administration of 6-OHDA toxin, and in such a case, a certain risk of fatal injury to the rats occurs. This is the reason why dopamine levels needed to be measured directly in the tissue in the present study.

### 2.7. Analytical Methods

Protein content was measured by the method of Lowry et al. [69].

Post-nuclear tissue homogenate was used to measure the following biochemical parameters: (a) Lipid peroxidation using a Lipid Peroxidation (MDA) Assay Kit (MAK085, Sigma-Aldrich, USA); (b) Level of total reduced glutathione using a Glutathione Assay Kit (CS0260, Sigma-Aldrich, USA); (c) Superoxide dismutase activity using an SOD determination kit (19160, Sigma-Aldrich, USA); (d) Activity of glutathione peroxidase using a Glutathione Peroxidase Cellular Activity Assay Kit (CGP1, Sigma-Aldrich, USA); and (e) Activity of catalase using the method of Aebi [70].

### 2.8. Statistical Analysis

Results were expressed as the mean ± S.E.M. Statistical analysis of the data were performed by using Student’s *t*-test for unpaired data or by one-way analysis of variance (ANOVA) followed by an post-hoc test (Duncan, Dunnett, or Newman-Keuls). Differences were considered significant at *p* < 0.05. 

#### Principal Component Analysis

Before investigation of the relationship between the studied variables, a standardization was applied through calculation of the Z-score for each variable (subtracting the standard deviation from the mean of the variable, and then dividing the difference by the standard deviation) [71]. Spearman’s correlation test was performed to assess correlation between studied parameters. Exploratory Principle component analysis (PCA) was applied on standardized data of 25 rats with values for all investigated variables for the detection of similarities and differences between the different samples, as well as the identification of the main associations between variables that are responsible for the total variability of the data studied. All calculations and graphs produced for the PCA study were performed using the software IBM SPSS 19. Hierarchical cluster analysis (centroid clustering method, squared Euclidean distance) was performed on extracted PCA component scores for the first two principal components in order to confirm clustering of the individual rats according to their treatment.

## 3. Results

### 3.1. Effect of EA, LA, and Myrt on Body Weight of Wistar Rats

**Figure 2:**
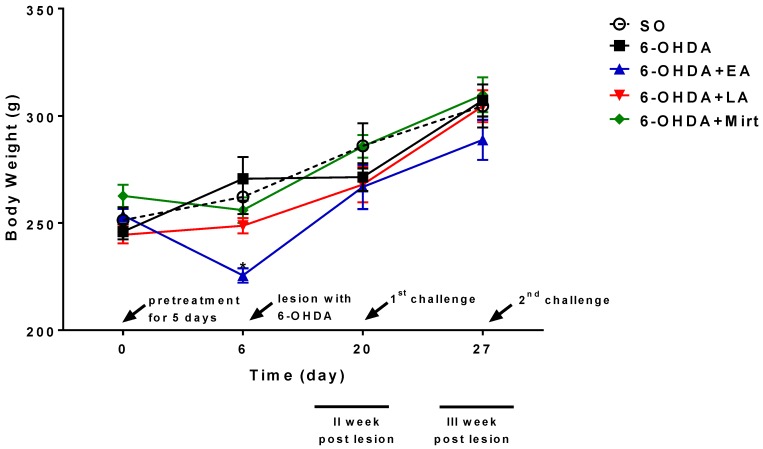
Effects of the EA, LA, and Myrt on body weight of the five groups of rats. The body weights (mean ± SEM) of each group of rats (*n* = 6–8 per group) were recorded. EA, LA, and Myrt (50 mg/kg, intraperitoneal [i.p.] injection) were administered daily for five days before stereotaxic surgery (unilateral injection of 2 μL/10 μg 6-hydroxydopamine [6-OHDA] into the right striatum). Asterisks indicate a significant difference in body weights between the experimental and 6-OHDA group at *p* < 0.01. Statistical analysis was by one-way analysis of variance (ANOVA) and Dunnett’s post hoc comparison test. For a detailed description of the groups, see section ‘Drug Treatment and Experimental Design’.

Pretreatment of experimental animals with test compounds resulted in body weight loss as compare to the control group. The most obvious effect was observed in elagic acid-treated animals where the mean body weight reduction was by 16% (*p* < 0.05) compared to the control. After suspension of daily administration of EA, LA, and Myrt, the body weights of the animals were increased, and this trend was maintained until the end of the experiment. The increased body weight of the tested animals is due to more intake of food. The results of the body weight measurements of the tested animals are summarized in Figure 2.

### 3.2. Behavioral Observation

#### 3.2.1. Apomorphine-Induced Rotations

**Figure 3:**
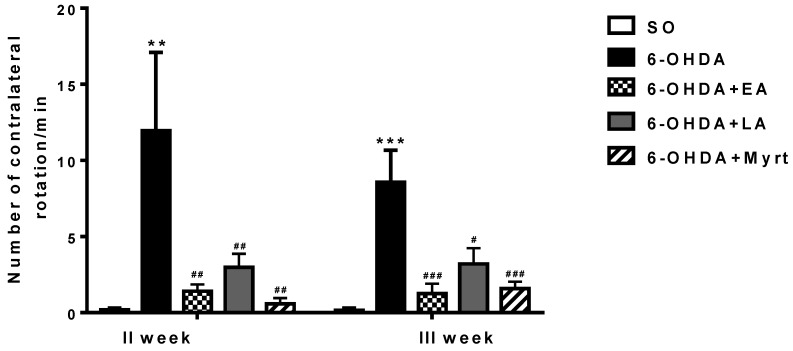
Effects of the EA, LA, and Myrt on the apomorphine-induced rotational behavior in a rat model of Parkinson’s disease (PD). EA, LA, and Myrt (50 mg/kg, intraperitoneal (i.p.) injection) were administered daily for five days before stereotaxic surgery (unilateral injection of 2 μL/10 μg 6-hydroxydopamine (6-OHDA) into the right striatum). The test was made at the II and III weeks post lesion. The animals were observed for 30 min, beginning 1 min after the apomorphine injection (2 mg/kg, i.p). Data are expressed as the mean ±standard error of the mean (SEM) (*n* = 6–8 animals per group). Asterisks above bars indicate a significant difference in the number of rotations per minute for the 6-OHDA group versus the sham-operated (SO) group at ** *p* < 0.01; *** *p* < 0.001. Hashtags above the bar indicate a significant difference in the number of rotations per minute for each experimental group versus the 6-OHDA group at # *p* < 0.05; ## *p* < 0.01; ### *p* < 0.001. Statistical analysis used one-way analysis of variance (ANOVA) and Dunnett’s post hoc comparison test. For a detailed description of the groups, see section ‘Drug Treatment and Experimental Design’.

In the 6-OHDA control group at the 2nd week post lesion, an average of 11.95/min of contralateral rotations was recorded. At the 3rd week, the average in this group was 8.6/min. The SO control group showed basically no evidence of rotation, indicating a lack of lesions (Figure 3).

Animals treated with EA, LA, and Myrt showed that the number of contralateral rotations/min was significantly reduced as compared to the 6-OHDA control group, with the best values for Myrt on week 2 (a reduction by 95.14%, *p* < 0.01, *n* = 8) and for EA on week 3rd (a decrease by 85.35%, *p* < 0.001, *n* = 8) post challenge. LA also decreased contralateral rotations but to a smaller degree by 75.1% on the 2nd week and by 62.76% on the 3rd week vs. 6-OHDA (Figure 3).

#### 3.2.2. Neuromuscular Coordination (Rotarod Test)

**Figure 4:**
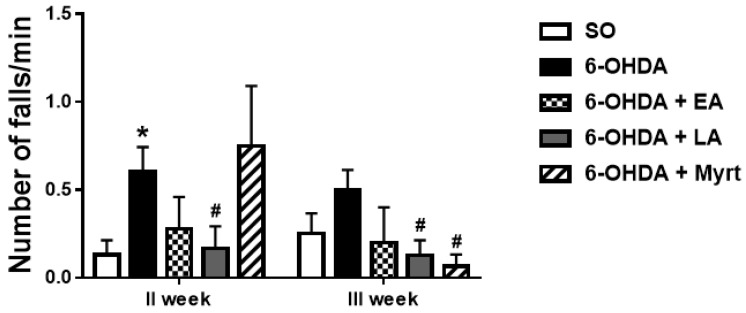
Effects of EA, LA, and Myrt on rotarod test in a rat model of PD. All substances (50 mg/kg, i.p.) were administered daily for five days before stereotaxic surgery (unilateral injection of 2 μL/10 μg 6-OHDA into the right striatum). The test was made at week II and week III post lesion. The animals were placed on a spinning bar (7 rpm/min), and the number of falls was determined for 1 min. Data are expressed as the mean ± SEM (*n* = 6–8 animals per group). Asterisks above bars indicate significant differences in the number of falls per minute between 6-OHDA and SO groups at * *p* < 0.05. Hashtags above bar indicate a significant difference in number of falls per minute between each experimental group and the 6-OHDA group at # *p* < 0.05. Statistical analysis was done by using Student’s *t*-test.

An average of 0.61 ± 0.14 and 0.5 ± 0.11 falls/min for the second and the third weeks post lesion, respectively, were observed in PD animals (Figure 4).

The animals treated with EA, LA, and Myrt showed improvements in neuromuscular coordination in the rotarod test, when compared to the control group treated with 6-OHDA. Maximum decrease in number of falls /min was achieved by LA (72.13%, *p* < 0.05, *n* = 8) for week 2 and by Myrt (86.8%, *p* < 0.05, *n* = 8) for week 3 after the lesion (Figure 4).

#### 3.2.3. Passive Avoidance Test

**Figure 5:**
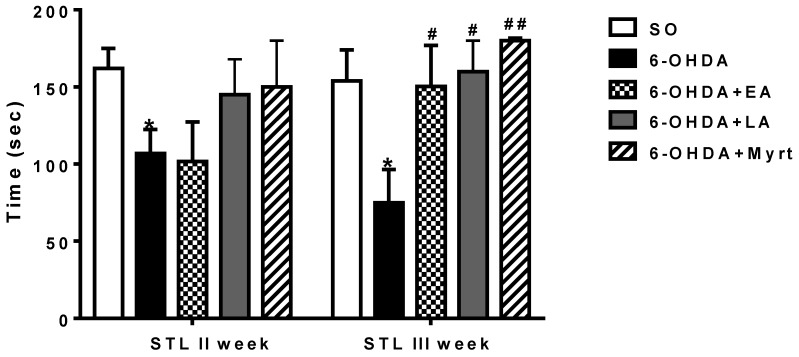
Effects of EA, LA, and Myrt on step-through latency (STL) in single-trial passive avoidance test in a rat model of PD. All substances (50 mg/kg, i.p.) were administered daily for five days before stereotaxic surgery (unilateral injection of 2 μL/10 μg 6-OHDA into the right striatum). The test was made at week II and week III post lesion. Data are expressed as the mean ± SEM (*n* = 6–8 animals per group). Asterisks above bars indicate a significant difference in STL between 6-OHDA groups and the SO group at * *p* < 0.05. Hashtags above bar indicate a significant difference in STL between the experimental group and the 6-OHDA groups at # *p* < 0.05; ## *p* < 0.01. Statistical analysis was done by using Student’s *t*-test.

Step through latencies (STL) of all groups in a single-trial passive avoidance test are shown in Figure 5. The memory performance in 6-OHDA control group was significantly impaired as compared to SO rats. In the former group, STL were reduced by 34.57% (*p* < 0.05, *n* = 8) and by 51.42% (*p* < 0.01, *n* = 8) on week 2nd and 3rd post lesion, respectively, vs. the latter (Figure 5). On the other hand, pretreatment of rats with the three AOs produced significant memory improvement. The SLT in these groups was longer with the best results during the second and the third weeks (post lesion) with Myrt increased by 40.44% and 140.61% (*p* < 0.01, *n* = 8), respectively. EA increased memory performance by 100% (*p* < 0.01, *n* = 8), and LA by 113.88% (*p* < 0.01, *n* = 8) vs. 6-OHDA on the 3rd week. The preventive effect of the three AOs became even more pronounced as observed on the 3rd week. It increased from the 2nd to the 3rd week for Myrt 3.5-fold (from 40.44% to 140.61%) and 3.9-fold for LA (from 35.76% to 113.88%). The improving memory effect of EA was remarkable—a 20-fold increase in the indicator values (up to 100%).

### 3.3. Modulation of Brain DA Levels by AOs Pretreatment

**Figure 6:**
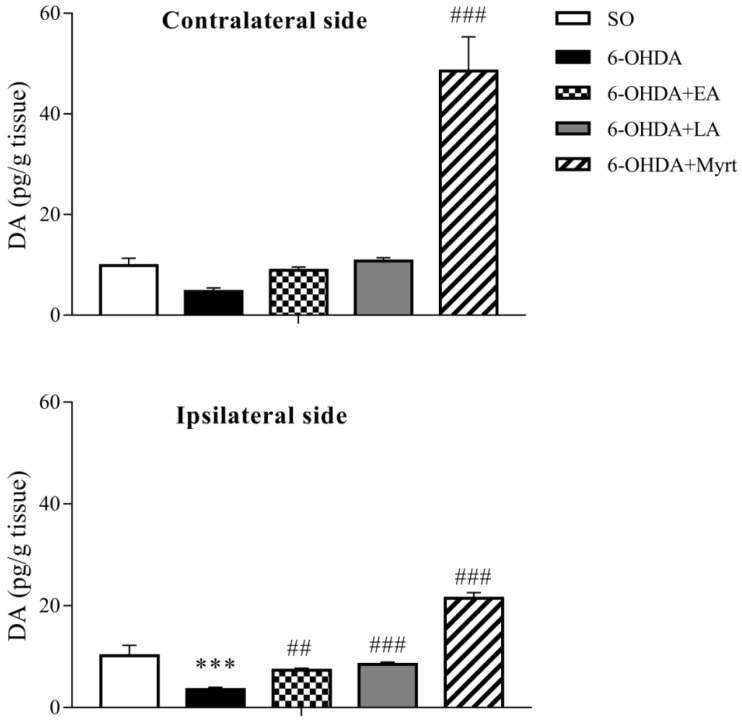
Effects of EA, LA, and Myrt (50 mg/kg, i.p.) on dopamine (DA) levels in ipsilateral (lesioned) and contralateral (intact) side of the brain. All test substances were administered daily for five days before stereotaxic surgery (unilateral injection of 2 μL/10 μg 6-OHDA into the right striatum). Data are expressed as the mean ± SEM (*n* = four animals per group). Asterisks above bars indicate significant differences in DA level between 6-OHDA and SO groups at *** *p* < 0.001. Hashtags above bars indicate a significant difference in DA level between each experimental group and the 6-OHDA group at ## *p* < 0.01; ### *p* < 0.001. Statistical analysis was performed by one-way ANOVA and Dunnett’s post hoc comparison test.

DA levels in brain homogenates were measured 21 days after neurotoxin application. In the 6-OHDA control group, DA levels were significantly decreased as compared to the SO group (Figure 6). Ipsilaterally the decrease was by 65.22% (*p* < 0.01, *n* = 8) and contralaterally the decrease was by 52.18% (*p* < 0.01, *n* = 8). 

In PD rats that had received EA, LA, and Myrt, the levels of DA in the brain were significantly increased as compared to the 6-OHDA group. DA levels were restored by EA by 108.9% (*p* < 0.01, *n* = 4) ipsilaterally and by 88.96% contralaterally. LA also increased this parameter by 140.23% (*p* < 0.001, *n* = 4) ipsilaterally and by 128.45% contralaterally, respectively vs. 6-OHDA control animals. 

The restoring effect on brain DA levels observed in LA and EA treated groups was stronger in ipsilateral (lesioned) than in contralateral (intact) side.

The best effect in terms of preserving DA brain levels was exerted by Myrt. The increase in this indicator was by 507.65% (*p* < 0.001, *n* = 4) ipsilaterally and by 929.93% (*p* < 0.001, *n* = 4) contralaterally, i.e., more than 5- and 9-fold, respectively, when compared to the 6-OHDA group. 

In contrast to LA and EA, Myrt possessed a stronger restoring effect on brain DA content on the contralateral side than on the ipsilateral side.

### 3.4. Effects of the Three Compounds on Parameters of Oxidative Stress in the Brain of PD Rats

**Figure 7:**
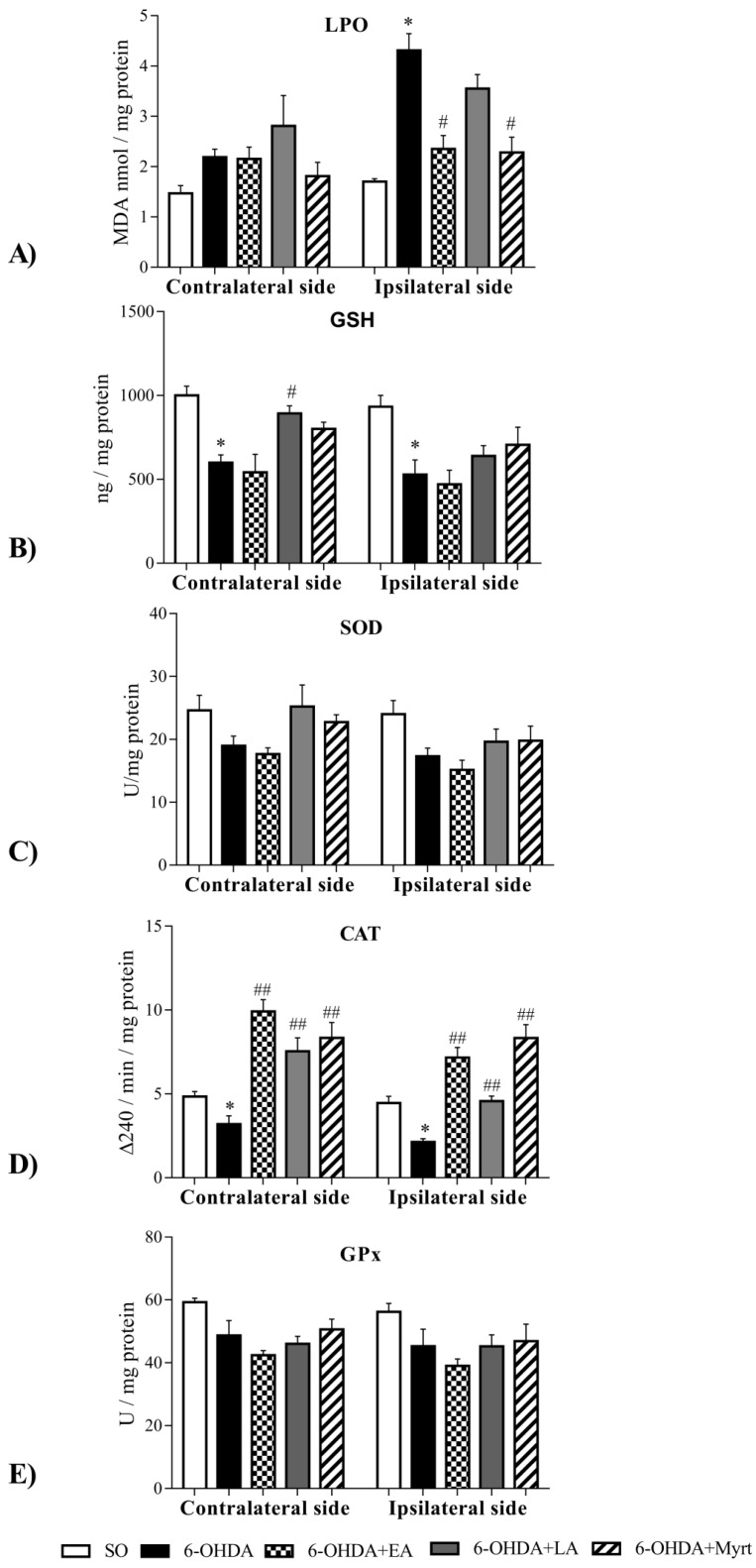
Effect of EA, LA, and Myrt (50 mg/kg, i.p.) on oxidative stress parameters: (**A**) lipid peroxidation; (**B**) total glutathione levels; (**C**) superoxide dismutase activity; (**D**) catalase activity and (**E**) glutathione peroxidase activity in brain homogenates of rats with a 6-OHDA model of PD. All tested substances were administered daily for five days before stereotaxic surgery (unilateral injection of 2 μL/10 μg 6-OHDA into the right striatum). Data are expressed as the mean ± SEM (*n* = four animals per group). Asterisks above bars indicate significant differences in parameters between 6-OHDA and SO groups at * *p* < 0.05. Hashtags above bars indicate a significant difference in parameters between 6-OHDA and SO groups at # *p* < 0.05; ## *p* < 0.01. Statistical analysis was performed by using Student’s *t*-test.

The treatment of animals with 6-OHDA leads to a significant increase of LPO levels, especially in the ipsilateral side of the brain (Figure 7A), as well as decreased levels of GSH (Figure 7B) and decreased catalase (CAT) activity (Figure 7D) both in ipsilateral and contralateral sides.

The activity of superoxide dismutase (SOD) (Figure 7C) and glutathione peroxidase (GPx) (Figure 7E) were also decreased, albeit insignificantly. 

The application of the tested substances partially restored the oxidative alterations induced by 6-OHDA (Figure 7). Thus, EA and Myrt reduced LPO in 6-OHDA treated rats only in the ipsilateral side by 46% (*p* < 0.05, *n* = 4) and 47% (*p* < 0.05, *n* = 4), respectively. In contrast, LA had no significant effect. As for the GSH level, LA increased the GSH brain level by 49%, *p* < 0.01, *n* = 4 (contralateral) and Myrt increased it by 34% in both hemispheres (contralateral and ipsilateral), while EA had no significant effect. In regard to CAT activity, the three tested compounds increased CAT activity in the brain, but showed hemisphere lateralization. The enhancing effect of EA and Myrt was more pronounced ipsilaterally (by 236% and by 291% respectively, *p* < 0.01, *n* = 4), whereas the effect of LA was weaker and more pronounced in contralateral side of the brain (by 136.36%, *p* < 0.01, *n* = 4). In regard to SOD, and GPx activity, EA, LA, and Myrt did not affect SOD and GPx activities significantly.

#### 3.4.1. Common Antioxidant Effects

Bioinformatics tools are gaining major attention for their use in several areas of biomedical research such as cancer and autoimmune diseases and help dismantle pathogenetic pathways and offer in silico prediction of theranostic molecules [72,73,74,75,76].

Principal component analysis (PCA) is a classic dimension reduction approach that constructs linear combinations of a set of observations, called principal components (PCs). The PCs are orthogonal to each other, can effectively explain variation of, e.g., gene expressions, and may have a much lower dimensionality [77].

PCA was presently employed to gain deeper understanding of the role played in induction of antioxidant effects by EA, LA, and Myrt, to identify which parameters of oxidative stress affect DA levels and behavior of the tested rats. Parameters of the three PD groups treated with EA, LA, and Myrt together with those of SO and PD untreated rats were analyzed. Principal components were calculated having all available data variables: (i) DA, GSH, and LPO levels; (ii) CAT, SOD, and GPx activities for contralateral and ipsilateral effects on the lesion side, (iii) relative brain weight, and scores of STL, RR, and Apo for the second and third week. The dataset was standardized in order to eliminate the effects of different magnitudes of the variables. The first five components were extracted in attempt to identify a potential component identifying each of the treated group. Table 1 shows that the first three principle components (PC1, PC2, and PC3) explained most of the observed variance (63%). This suggests the existence of strong patterns of the correlation between some of the experimental variables. Further investigation of PC1 alone (explains 29% of the variance) showed a strong positive relationship between DA levels and the majority of the measures of the oxidative stress both at contra and ipsilateral sites (Table 1). The only exception here is lipid peroxidation, which is negatively correlated specifically in the lesioned locations (Table 1). On the other hand, PC2, which explains 23% of the variances, relates positively levels of lipid peroxidation in the lesioned side toward other parameters of oxidative stress (GSH, CAT, SOD, and GPx) and with behavioral performance. PC3 (11% of the variance) demonstrates association of DA levels with memory and motor performance (RR and Apo). Together, PC4 and PC5 explained a relatively small amount of the variance of the parameters (around 15%) that is why we discarded them from further interpretation. Thus, we can conclude that there might be three major mechanisms related to antioxidant recovery in PD rats. AOs increase dopamine levels in the brain and reduce lipid peroxidation in the lesioned regions (PC1), independently changes of oxidative stress are related to the behavioral parameters (PC2) and finally dopamine levels showed association with behavioral performance (PC3). This observation implies that common antioxidant protection in PD rats depends on three distinct mechanisms of recovery.

#### 3.4.2. Specific Antioxidant Effects

**Figure 8:**
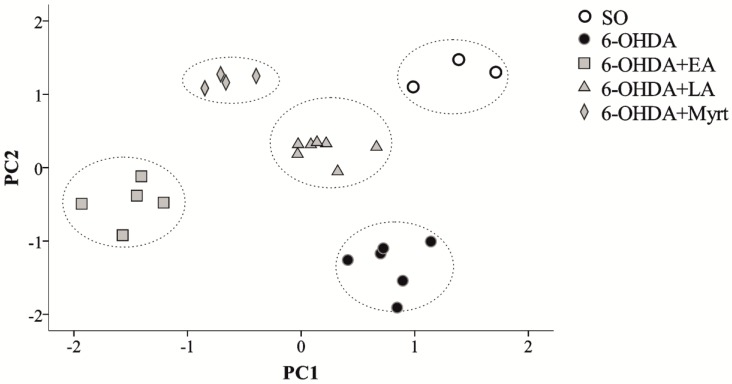
Representation of the individual rat data points, using the first two principal components (PC1 and PC2) on a two dimensional space, shows that the rats tend to cluster according to their treatments. Hierarchical cluster analysis of the data (dotted ellipses). SO—white circles; PD 6-OHDA—black circles, 6-OHDA + EA—grey squares, 6-OHDA + LA—grey triangles, 6-OHDA + Myrt—grey diamonds.

To investigate whether different mechanisms are involved in the effect of the three examined compounds, we performed a principal component analysis (PCA) on a set of variables including DA, LPO, GSH, CAT, SOD, and GPx levels on ipsilateral and contralateral relationships to the 6-OHDA injection site together with the brain weight, Rota-rod, and Apomorphine test scores at the second and third week, respectively. After initial analysis, a total of five principal components were identified (PC1–PC5). After close inspection of the scree plot and variance explained by the PCs, a second PCA was run with fixed number of factors equal to two. It turned out that those two—PC1 and PC2—explained 54 percent of the total variance and the Kaiser-Meyer-Olkin criterion was higher than 0.6, which verified that sampling was adequate. Major contributors (factor loadings greater than 0.3) to PC1 showed a strong relation between DA levels and oxidative stress as reflected by GSH, CAT, SOD, and GPx but not LPO levels at both sites. PC2 demonstrated a relationship between the rat’s cognitive performance (Apomorphine test at second and third week and Rota-rod at third week) and oxidative stress (LPO at ipsilateral locations and GSH, SOD, and GPx at ipsilateral and contralateral locations). Figure 8 represents a 2D scatter plot produced by factor loadings of PC1 and PC2 calculated for each of the 25 examined animals. There is a strong rat tendency to cluster together according to their treatments. As confirmation, Figure 8 further shows that hierarchical cluster analysis on the calculated PC1 and PC2 scores correctly grouped and isolated the data points from all different treatments (doted ellipses). Thus, statistically we can say that AOs groups are clearly separated from untreated PD rats and their proximity to the SO controls. Furthermore, it is evident quantitative distinction within the AO treatments, which imply at least partial differential mechanisms of PD protection by EA, LA, and Myrt. 

## 4. Discussion

Whilst the etiology of PD remains unclear so far, numerous reports suggest a connection between OS and the pathogenesis of the disease [78,79]. One of the concepts of PD pathogenesis focuses on the formation of ROS and the role of oxidative stress leading to damage of SNPC neurons. This pathogenetic hypothesis is supported both by preclinical studies in animal models of PD [80] and extensive postmortem studies in PD patients, where it has been observed to have caused impaired mitochondrial function, alterations in antioxidant protective systems (most notably superoxide dismutase and reduced glutathione), extensive oxidative damage to lipids, proteins, and DNA [81].

This converging evidence indicating a key role of OS in PD propel the search for appropriate antioxidants to neutralize oxidative processes. However, treatment with common antioxidants such as vitamin C, vitamin E, and coenzyme Q10 exhibited poor effects in disease delay. For this reason, the search for either synthetic or other natural substances with rich biological properties and complex multi-target mechanisms continue for effective therapy or the prevention of PD [82,83].

The antioxidants EA and LA have been investigated as a new therapeutic alternative for neurodegenerative disorders such as AD and PD, as well as for depression, ischemia, and other disorders [84,85,86].

Our previous studies also established the significant preventive effect of EA and LA as well as of Myrt on experimental scopolamine (Sco) induced dementia from types of Alzheimer’s disease in rodents. We have proved that neuroprotective, neuromodulatory, and improving learning and memory effects, accompanied by strong AO activity allow multi-target treatment of the neurodegenerative process [25,40,43,57,87].

The 6-OHDA hemilesioned PD rat model that we have presently used is a well-recognized in vivo tool to investigate mechanism and therapeutic strategies for PD treatment. 

The neurotoxin 6-OHDA is a structural analogue of dopamine and noradrenaline and possesses high affinity for several catecholamine-ergic plasma membrane transporters (DAT and NAT respectively), which recognize and uptake 6-OHDA, which is a highly active dopaminergic neurotoxin that disrupts the catecholamine transport system and induces neurotoxicity by selective generation of OS and neuroinflammation in basal ganglia [88]. In addition, 6-OHDA is toxic both at a peripheral and central level. However, since the neurotoxin is incapable of crossing the blood-brain barrier, its toxicity in the CNS is achieved only when directly injected into the brain by stereotaxic surgery. The classical method of intracerebral infusion 6-OHDA involves a massive destruction of nigrostriatal dopaminergic neurons and is largely used to investigate motor and biochemical dysfunctions in Parkinson’s disease [89,90]. The neurotoxin causes selective and massive loss of dopaminergic neurons via generation of oxidative stress [91,92,93,94], mitochondrial dysfunction [95], and neuroinflammation with microglial activation [96].

The so-induced model mimics PD-like motor deficits relatively reliably and can partly recapitulate the progression of the PD pathology [97].

In this study, several PD-like behaviors were comprehensively evaluated using the model, including: the apomorphine-induced rotational behavior, which is related to the degree of nigrostriatal DA loss and is considered to be the “gold standard” in the hemilesioned rat model [97]; the rotarod test which is often used to appraise the balance skills and motor co-ordination in the PD rats [98]; and the step through test often used to evaluate learning and memory performance in PD animals. The PD model establishment worked clearly well, as the behavioral performance of the PD rats was impaired compared to in SO rats. In particular, 6-OHDA produced an increase in the number of contralateral rotations induced by apomorphine. The latter is a DA receptor agonist that at low doses causes contralateral turning by stimulating both supersensitive D1 and D2 receptors preferentially on the denervated side [99]. The neurotoxin treatment also increased the number of falls/min in rotarod test and decreased STL in a step-trough test.

Our experimental data showed that the three tested substances decreased significantly the number of contralateral rotation in apomorphine test with the best effect being observed 3 weeks post lesion for EA and decreased the number of falls/min in rotarod test with best effect for Myrt, 3 weeks after lesion. This result confirmed the reported improvement in motor coordination effect of EA and decreased the contralateral rotation effect of LA in 6-OHDA-induced neurotoxicity on a rat model of hemi-parkinsonism [87,100]. To the best of our knowledge, no studies have so far been conducted with Myrt on PD models. The data from our behavioral test showed that EA, LA, and Myrt had comparable neuroprotective effects against 6-OHDA induced neural oxidative damage.

In addition, particularly interesting results were obtained from the step through test, where alteration in learning and memory performance was evaluated by the change in STL time. In our experiment, we found that all three tested substances exhibited very good memory-enhancing effects, with the best result again for Myrt at three weeks post lesion. It is well known that learning and memory processes are primarily dependent on the cholinergic neurons in the hippocampus, cerebral cortex, and some parts of the new striatum [101]. The ability of EA to regulate cognitive function through inhibition of the acetylcholinesterase (AChE) activity (IC50 = 13.79 μg/mL) and through acetylcholine upregulation was already reported [102]. A memory-enhancing effect and increased acetylcholine (ACh) level in the brain of dement animals after Myrt treatment (in combination with LA) was indicated for the first time in our data that was reported previously [57]. The AChE inhibitory effect of Myrt was only established in vitro in 2011 [59]. 

Damage and loss of dopaminergic neurons in the SNPC lead to decreased DA levels in the neostriatum, which underlies the symptoms of Parkinson’s disease [103]. Therefore, we detected DA level in the brain in this study, and as expected, the results showed that the impaired behavior were due to the depletion of DA in the striatum in PD- rats in comparison to SO group. The three tested compounds, Myrt, EA, and LA, restored DA brain levels in the ipsilateral and contralateral side, but showed hemisphere lateralization. The strongest (more than 10-fold vs. the 6-OHDA group) was the effect of Myrt and this effect was more pronounced in the contralateral side of the brain. The effects of EA on DA were weaker than that of LA and for both, the observed effects were stronger in ipsilateral (lesioned) side of the brain. The ability of LA to restore DA levels was reported in 6-OHDA- and MPTP-induced models of PD [104]. In our previous studies with Sco-induced model of dementia, we have established that Myrt significantly increases DA levels in the brain [87]. The ability of the tested AOs to increase dopamine levels in the brain after the neurotoxin action (6-OHDA) provides additional strong in vivo proof-of-concept of their neuroprotective properties. In addition to pure neuronal preservation, our results hinted at a possible neuromodulatory effect, which was especially evident for Myrt.

In the present study we confirmed the relation between neuronal degeneration in 6-OHDA model of PD and OS as a main pathogenic factor in disease progression [4,6,105]. Oxidation of 6-OHDA by molecular oxygen or monoamine oxidase underlies its neurotoxicity in the brain and leads to production of intracellular H_2_O_2_ which can be transformed into highly reactive hydroxyl radicals, a reduction in glutathione (GSH) and SOD activity, an increase in LPO and production of superoxide free radicals causing cell damage [106]. A decrease of GSH in SNPC is the earliest known indicator of oxidative stress in pre-symptomatic PD, which precedes decreases in dopamine levels [81].

Our results established increased LPO and reduced glutathione levels in PD- brain and this alteration was available both in the ipsilateral and contralateral sides. In agreement with this post-mortem analyses and further strengthening the preclinical relevance of the 6-OHDA model of PD presently used, we also observed a significant decrease in activities of main AO enzymes (SOD, CAT and GPx) in PD rats in comparison to the SO group (Figure 7). Along with this, the administration of the three tested bio-AOs, Myrt, LA, and EA for five consecutive days demonstrated that:

1. The three bio- AOs decreased LPO levels, ipsilateral in the brain and the strength of the effect diminished in the following sequence EA = Myrt > LA;
2. LA and Myrt increased GSH brain levels. The effect of the dithiol compound was better and contralaterally located. The monoterpenoid Myrt enhanced GSH both ipsilateral and contralateral effects in the same way.

Altogether, it could be suggested that the use of Myrt, LA, and especially EA, alone or in combination may contribute to alleviate the progression of PD.

All tested substances increased CAT activity. Increased CAT activity by EA and Myrt was more pronounced ipsilaterally and by LA — contralaterally. The most powerful effect in this setting was that of Myrt. In our experimental conditions, we did not establish significantly increased SOD activity to be caused by EA, as reported by Sarkaki et al. [86]. This difference may be due to different experimental condition. We applied EA before lesion, while Sarkaki et al.—after lesion [86]. It can be concluded that all three tested compounds enhanced the cerebral antioxidant defense with different strength and hemispheric specificity, but all led to a reduction of oxidative stress. 

While on the one hand we confirmed previous data on neuroprotective action exhibited by EA and LA against 6-OHDA-induced neural oxidative damage [86,100], on the other we demonstrate the powerful pharmacological effects of Myrt towards experimental PD here for the first time.

We also established statistically some common antioxidant mechanisms of PD recovery in rats in two major brain areas [4]. There is a direct relationship between levels of oxidative stress and function of the DA neurons in PD animals. A direct correlation between DA system recovery and improvement in motor performance by AO in rats was established. Decrease of oxidative stress and particularly lipid peroxidation was directly associated with improved behavioral performance in AO treated animals. The respective data are presented in Table 1.

In order to distinguish general from specific mechanisms of the neuroprotective action of the three natural antioxidants used, we performed a cluster analysis where we used the principal component scores derived from a set of biochemical data (DA levels together with OS parameters) and behavioral parameters (motor coordination and memory) (Figure 5). Cluster analysis correctly grouped and isolated the data points from all different treatments (SO, 6-OHDA, EA, LA, Myrt). It correctly distinguishes the 6-OHDA (black dots) from other treatments. This approach provided quantitatively supports two conclusions:

(1) Significant PD recovery by the three AOs—there is clear evidence for neuroprotective AO effects and differentiation of the treated groups from PD rats.
(2) There are close but yet distinguishable mechanisms of neuroprotection for each of three AOs: LA, EA, and Myrt.

Observed specific PD—the preventive effect of EA, LA, and Myrt was related to their diverse rich biological activity. The improving memory effect of EA probably is specific and was due to its active urolithins metabolites, which can easily penetrate the blood–brain barrier [107]. The recovering memory effect of LA in a mouse model of AD type dementia [40,43] is related to direct or indirect AO effect of LA [58,63,85] and to neuromodulatory activity of Myrt [26,40,87].

On the basis of the present experiment, we presumed that under the similar neurodegenerative effects of the three AOs lay down different mechanisms related to their powerful biological activity. This could explain why in spite of their different chemical structure and specific biologic activities, the three compounds could effectively prevent the neurodegenerative process in PD animals using their diverse multi-target capacities.

In this regard, future studies with combined use of Myrt, LA and EA in different combinations may prove useful to evaluate whether these compound specific-effects and their hemispheric specificity may be synergistically complemented by their combined use. If Myrt, EA, and LA exert synergistic effects in vivo, lower doses of each compound may afford an even stronger anti-Parkinsonian effect than when they are applied individually.

## 5. Conclusions

Our comparative study indicated that all tested bio-AOs possessed powerful neuroprotective effects in the 6-OHDA-induced PD model in rats via common AO mechanisms. Their ability to increase dopamine levels in the brain and to enhance the cerebral antioxidant defense (with different strength and hemispheric specificity) leads to a reduction of oxidative stress. 

Cluster analysis also confirmed a significant delay in PD progression by the three AOs but it also strongly suggested specific neuroprotective effects of LA, EA, and Myrt due to their rich and complex biological activities. 

For the first time a significant anti-Parkinsonian effect of Myrt was reported in our work. Myrt possessed the strongest protective effect among the studied AOs—as evaluated by behavioral and biochemical parameters in the brain. 

Brain laterization in the damaging effect of the neurotoxin 6-OHDA, and in the protective action of the three compounds was found. In regard to their antioxidant properties, EA and Myrt affect strongly the ipsilateral brain side (the lesioned side) while LA predominately affected the contralateral (healthy) brain side. In regards to their DA recovery properties, the effect of EA and LA was more pronounced ipsilaterally, whereas Myrt had more of an effect contralaterally.

Our data overall demonstrate the complexity of the neurodegenerative process and the real possibility that it could be affected preventively by multi-target strategies.

## Figures and Tables

**Table 1:** The first five principal components scores instructed by PCA.

Parameters ^1^	PC1	PC2	PC3	PC4	PC5
DA-L	0.60	−0.34	0.64	−0.01	−0.18
DA-D	0.69	−0.37	0.49	0.06	−0.11
LPO-L	−0.29	−0.18	0.04	0.76	0.21
LPO-D	−0.72	0.46	0.27	0.40	0.02
GSH-L	0.72	0.54	−0.14	0.32	0.01
GSH-D	0.74	0.48	−0.07	−0.03	0.14
CAT-L	0.31	−0.86	−0.29	0.11	−0.11
CAT-D	0.57	−0.79	0.02	−0.09	−0.08
SOD-D	0.54	0.45	−0.23	0.49	−0.26
SOD-L	0.56	0.58	−0.05	0.24	−0.34
GPx-L	0.61	0.58	0.16	−0.26	0.19
GPx-D	0.29	0.76	−0.18	−0.36	−0.10
Brain Weight	−0.35	−0.32	−0.67	0.15	0.11
RR-Week 2	0.12	−0.11	0.74	0.31	0.23
RR-Week 3	−0.52	0.31	0.18	−0.36	−0.07
Apo-Week 2	−0.58	0.38	0.18	0.15	0.19
Apo-Week 3	−0.68	0.40	0.32	−0.01	−0.32
Component coefficients	5.51	4.39	2.15	1.71	1.15
Variance explained	29.01	23.09	11.31	8.98	6.04

^1^ Principal component analysis (PCA) factor loadings, variance explained, and coefficients for all investigated variables are presented. On the left is the list of variables, where L stands for the contralateral side and D for ipsilateral side of the lesion; performance results are shown for the second and third week, respectively. Factor loadings greater than 0.3, are represented in bold.
